# G-OnRamp: Generating genome browsers to facilitate undergraduate-driven collaborative genome annotation

**DOI:** 10.1371/journal.pcbi.1007863

**Published:** 2020-06-04

**Authors:** Luke Sargent, Yating Liu, Wilson Leung, Nathan T. Mortimer, David Lopatto, Jeremy Goecks, Sarah C. R. Elgin

**Affiliations:** 1 Department of Biomedical Engineering, Oregon Health & Science University, Portland, Oregon, United States of America; 2 Department of Biology, Washington University in St. Louis, St. Louis, Missouri, United States of America; 3 School of Biological Sciences, Illinois State University, Normal, Illinois, United States of America; 4 Department of Psychology, Grinnell College, Grinnell, Iowa, United States of America; University of Toronto, CANADA

## Abstract

Scientists are sequencing new genomes at an increasing rate with the goal of associating genome contents with phenotypic traits. After a new genome is sequenced and assembled, structural gene annotation is often the first step in analysis. Despite advances in computational gene prediction algorithms, most eukaryotic genomes still benefit from manual gene annotation. This requires access to good genome browsers to enable annotators to visualize and evaluate multiple lines of evidence (e.g., sequence similarity, RNA sequencing [RNA-Seq] results, gene predictions, repeats) and necessitates many volunteers to participate in the work. To address the technical barriers to creating genome browsers, the Genomics Education Partnership (GEP; https://gep.wustl.edu/) has partnered with the Galaxy Project (https://galaxyproject.org) to develop G-OnRamp (http://g-onramp.org), a web-based platform for creating UCSC Genome Browser Assembly Hubs and JBrowse genome browsers. G-OnRamp also converts a JBrowse instance into an Apollo instance for collaborative genome annotations in research and educational settings. The genome browsers produced can be transferred to the CyVerse Data Store for long-term access. G-OnRamp enables researchers to easily visualize their experimental results, educators to create Course-based Undergraduate Research Experiences (CUREs) centered on genome annotation, and students to participate in genomics research. In the process, students learn about genes/genomes and about how to utilize large datasets. Development of G-OnRamp was guided by extensive user feedback. Sixty-five researchers/educators from >40 institutions participated through in-person workshops, which produced >20 genome browsers now available for research and education. Genome browsers generated for four parasitoid wasp species have been used in a CURE engaging students at 15 colleges and universities. Our assessment results in the classroom demonstrate that the genome browsers produced by G-OnRamp are effective tools for engaging undergraduates in research and in enabling their contributions to the scientific literature in genomics. Expansion of such genomics research/education partnerships will be beneficial to researchers, faculty, and students alike.

## Author summary

Major projects now underway aim to sequence most of the multicellular organisms on earth (e.g., the Earth Biogenome Project). But obtaining this data is only the beginning. To understand these organisms and how they relate to each other, we need to annotate their genomes (i.e., identify the genes and other features). While computers are essential for this process, most annotation tasks still require or benefit from human analyses. Genome browsers allow annotators to quickly visualize and evaluate multiple lines of evidence to create the best gene models. Hence, annotation of large number of eukaryotic species requires efficient generation of genome browsers and recruitment of many volunteers to participate. We have previously developed a web-based platform (G-OnRamp) to reduce the technical barriers for creating genome browsers. Using the G-OnRamp browsers, we engaged 15 faculty and their students in a Course-based Undergraduate Research Experience (CURE) focused on genome annotation of parasitoid wasp species. We find that G-OnRamp browsers work well in the classroom, and these efforts are beneficial for students and researchers. Students gain research experience, learn about genes and genomes, and learn how to work with large datasets. Researchers obtain high-quality datasets that could not be generated in any other way.

## Introduction

### The need for G-OnRamp

A considerable effort has been made over the last two decades to improve undergraduate science education by engaging students in the process of science, as well as acquainting them with the resulting knowledge base. For the life sciences, these efforts were perhaps best enunciated by the American Association for the Advancement of Science (AAAS) report *Vision and Change in Undergraduate Biology Education* [1]. One of the strategies found to be effective in engaging large numbers of undergraduates in doing science is the Course-based Undergraduate Research Experience (CURE [2]; see [3] and [4] for examples). Within computational biology, a number of groups have found that genome annotation is a research problem that can be adapted to this purpose.

With the decreasing cost and wide availability of genome sequencing [5], the bottleneck for utilizing genomics datasets to address scientific questions is shifting from the ability to produce data to the ability to analyze and interpret data. Genome annotation—labeling functional regions of the genome such as gene boundaries, exons, and introns—benefits from a combination of computational and manual curation of data. With appropriate tools and training, undergraduates can make a significant contribution to a community annotation project, where scientists work together to annotate part or all of a genome. Gene annotation builds on what students are learning about gene structure, while requiring them to grapple with multiple lines of evidence to establish defendable gene models. Student annotation projects thus are mutually beneficial for researchers and for students, enabling unique science and providing a multifaceted learning experience for students [6–10].

However, despite the improvements in tool accessibility and quality, there remain technical barriers that must be overcome to perform genome annotation. Many biology researchers and educators lack detailed knowledge of informatics and computational tools. When these scientists acquire the genome assembly of their favorite eukaryotic organism, one such technical barrier is the need to use multiple bioinformatics tools to analyze the genome assembly and visualize the results in a genome browser—the display tool central to community annotation. There are several good options, but most either require substantial computer skills and bioinformatics expertise to use, or have compute and storage limits that restrict the size/complexity of genome assemblies that can be analyzed using the platform [11–15].

We developed G-OnRamp to address these concerns. G-OnRamp is a collaboration between the Galaxy project (https://galaxyproject.org/), an open-source, web-based computational workbench for analyzing large biological datasets [16], and the Genomics Education Partnership (GEP; http://gep.wustl.edu/) [8,17]. Among G-OnRamp’s principal goals is lowering technical barriers to enable biologists to construct either a UCSC Assembly Hub [18] or a JBrowse/Apollo genome browser [19]. G-OnRamp accomplishes this by providing a collection of tools, workflows, and services preconfigured and ready to process data and enable annotation [20]. Students, educators, and researchers can bypass most of the system administration tasks involved in generating a genome browser and focus on using the genome browser to address scientific questions. Our assessment results in the classroom demonstrate that the genome browsers produced by G-OnRamp are effective tools for engaging undergraduates in research and in enabling their contributions to the scientific literature in genomics.

## Results

### Overview of the components

#### Genome annotation needs for the GEP

**Fig 1:**
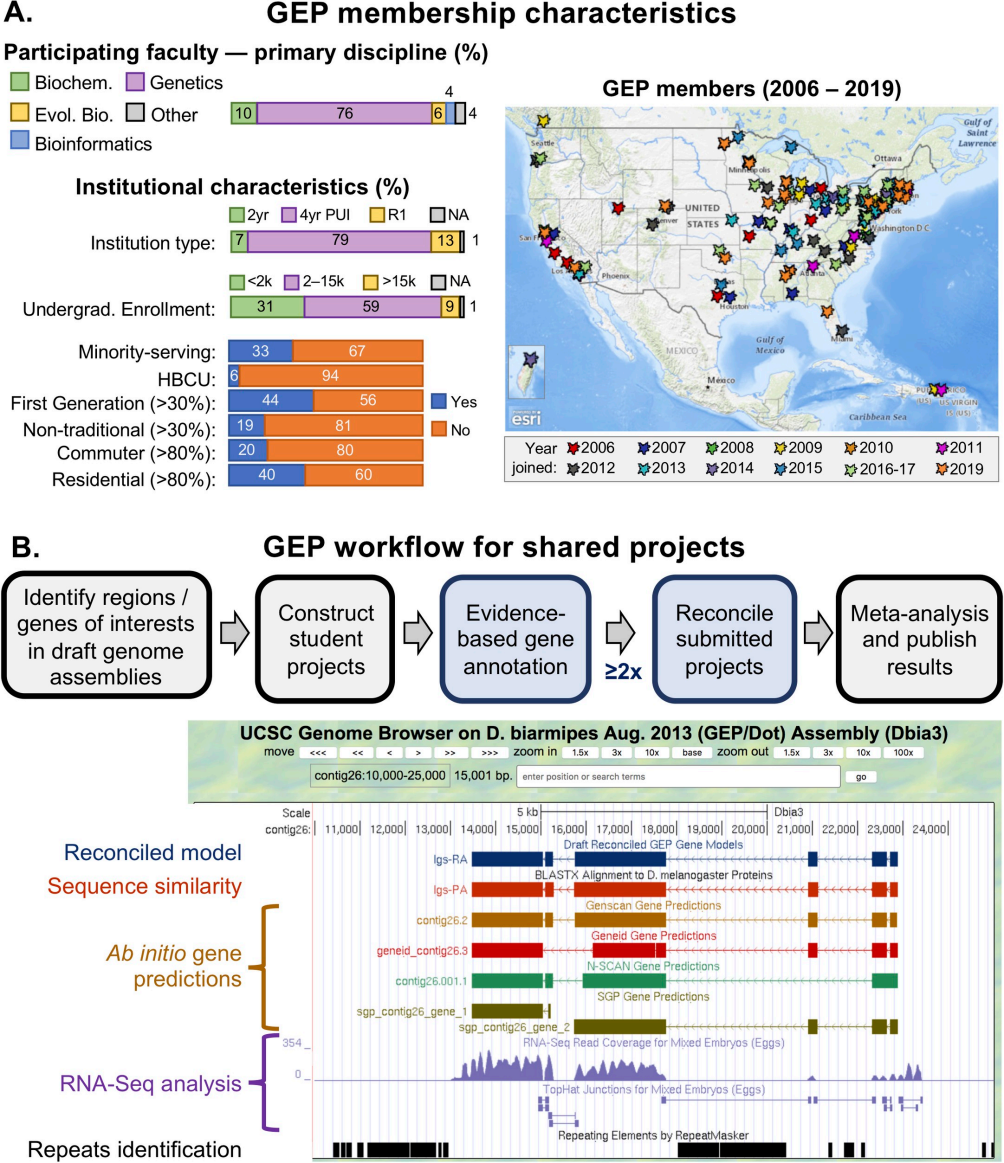
Overview of the GEP. A. Membership characteristics: participating faculty primarily teach genetics (although other disciplines are represented) and most often teach at primarily undergraduate institutions (PUIs) across the United States; faculty at community colleges and R1 research universities also participate. The geographical distribution of member schools and year of joining GEP are shown on the map. The member schools serve a diverse undergraduate student body, with 33% Minority-Serving Institutions (MSIs), including six Historically Black Colleges and Universities (HBCUs); 44% of the schools have 30% or more first-generation students, 11% have 30% or more nontraditional students (over 25 years of age), and 20% are commuter schools, with over 80% of the students commuting. See the Current GEP Members page (http://gep.wustl.edu/community/current_members) for a complete list of participating faculty with their schools. Map services and data available from the US Geological Survey, National Geospatial Program. B. Students in the GEP work together to produce high-quality annotation of a genome region or a collection of genes of interest identified by a Science Partner. “Student projects” are provided as genome browser pages (see lower portion of the figure), with one to seven potential genes (and other features of interest) for annotation. Browser tracks show available evidence for a gene, including gene conservation (sequence similarity track and additional BLAST searches), the presence of large open reading frames and other appropriate signals (ab initio gene predictions), and evidence of gene expression (RNA-Seq data, TopHat analysis results, etc.). Students work from these multiple lines of evidence, some of which may initially appear contradictory, to generate a gene model that they can defend. In the case shown, the sequence similarity search (BLAST) failed to identify putative upstream exons, whose presence is supported by RNA-Seq data and TopHat analysis. Students take responsibility for the workflow steps shown in light blue, while the Science Partner’s research group is responsible for the steps shown in gray. Pre-/post-course assessment has shown the effectiveness of such a collaborative annotation project both for supporting student learning about genes and genomes and in providing a research experience [17,21,22]. Biochem, Biochemistry; Evol. Bio., Evolutionary Biology; GEP, Genomics Education Partnership; RNA-Seq, RNA sequencing.

The GEP is a consortium of faculty members from over 100 educational institutions, which annually introduces more than 1,300 undergraduates to genomics research through engagement in collaborative annotation projects (Fig 1A). The GEP core organization provides technical infrastructure as well as identifying research questions that would benefit from high-quality gene annotations, particularly those for which utilizing comparisons across multiple species can provide insights. By engaging the talents of “massively parallel undergraduates,” one can gather data (high-quality annotations of hundreds of genes) that could not be obtained otherwise, given the limited number of domain experts and the amount of time and labor required to perform these analyses. To ensure that the gene annotations are high quality, each gene is annotated by at least two students working independently, and the results are reconciled by experienced students (Fig 1B).

**Fig 2:**
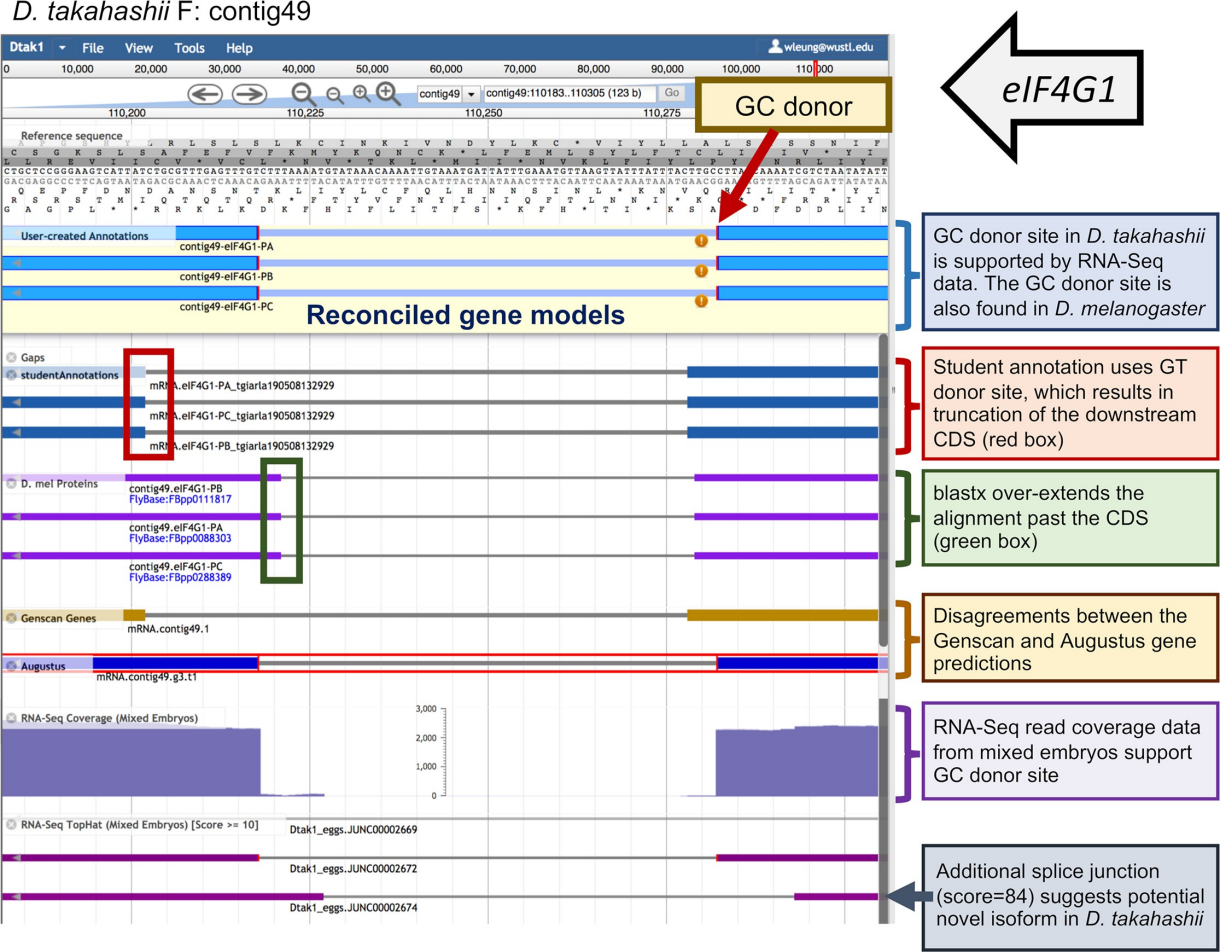
Apollo overview. After uploading data to Apollo via G-OnRamp’s "Create or Update Organism" tool, a user can choose which tracks to display with computational and experimental evidence, including submitted annotations from students, and begin to create her own gene model in a user-created annotations panel. Pictured is the Apollo interface showing provided sample data and computed lines of evidence, in addition to student annotation data and the final reconciled gene models (shown in the user-created annotations panel). The genome browser image illustrates a typical error by one student annotator at an intron/exon boundary. The standard protocol requires a minimum of two independent student submissions, followed by reconciliation by an experienced student annotator. Based on RNA-Seq data and the use of the noncanonical GC donor site in the informant species (*Drosophila melanogaster*), the reconciled gene model for the *D*. *takahashii* ortholog of *eIF4G1* uses a noncanonical GC splice donor site instead of the GT donor site proposed by the student annotator. CDS, codon sequence; RNA-Seq, RNA sequencing.

These collaborative genome annotation projects can be performed by students using either a genome browser or a genome annotation editor such as Apollo. Pedagogically, there are advantages to requiring students to initially examine the evidence tracks on a genome browser, using the data to determine the precise exon coordinates for their gene model, and recording the results in an Excel worksheet or other table. These models can then be imported into the genome browser as custom tracks and used as evidence in the final reconciliation. Currently, the GEP uses a hybrid approach, whereby students in GEP courses use a UCSC Genome Browser to construct the initial gene models, while experienced students use the Apollo annotation editor for finale reconciliation, using submitted student gene models as additional evidence tracks. The student reconcilers work under the direct supervision of the GEP Science Partner who initiated the project and will use the reconciled gene models in a meta-analysis (Fig 1B). See Fig 2 for an example of a typical error in a gene model submitted by a GEP student, viewed in Apollo for reconciliation. Overall, we see complete agreement in 60%–80% of the models submitted, depending on the difficulty of the project.

GEP faculty have worked collaboratively to generate and maintain curricula to introduce students to the appropriate computer-based tools and to the scientific questions under study [8,21]; all such materials are available on the GEP website under a “creative commons” license. Students who contribute documented gene models and participate in reading and critiquing the final manuscript are coauthors on the resulting scientific publication based on meta-analysis using these gene models (e.g., [23,24]). The gene annotations are submitted to GenBank as part of the publication so that they are available for use by other researchers. G-OnRamp was conceived by the GEP as a component of the technical infrastructure, simplifying the process of generating genome browsers. This capability should allow biology faculty to diversify the research questions under study, exploiting newly sequenced genomes as they become available.

#### G-OnRamp tools and workflows

**Fig 3:**
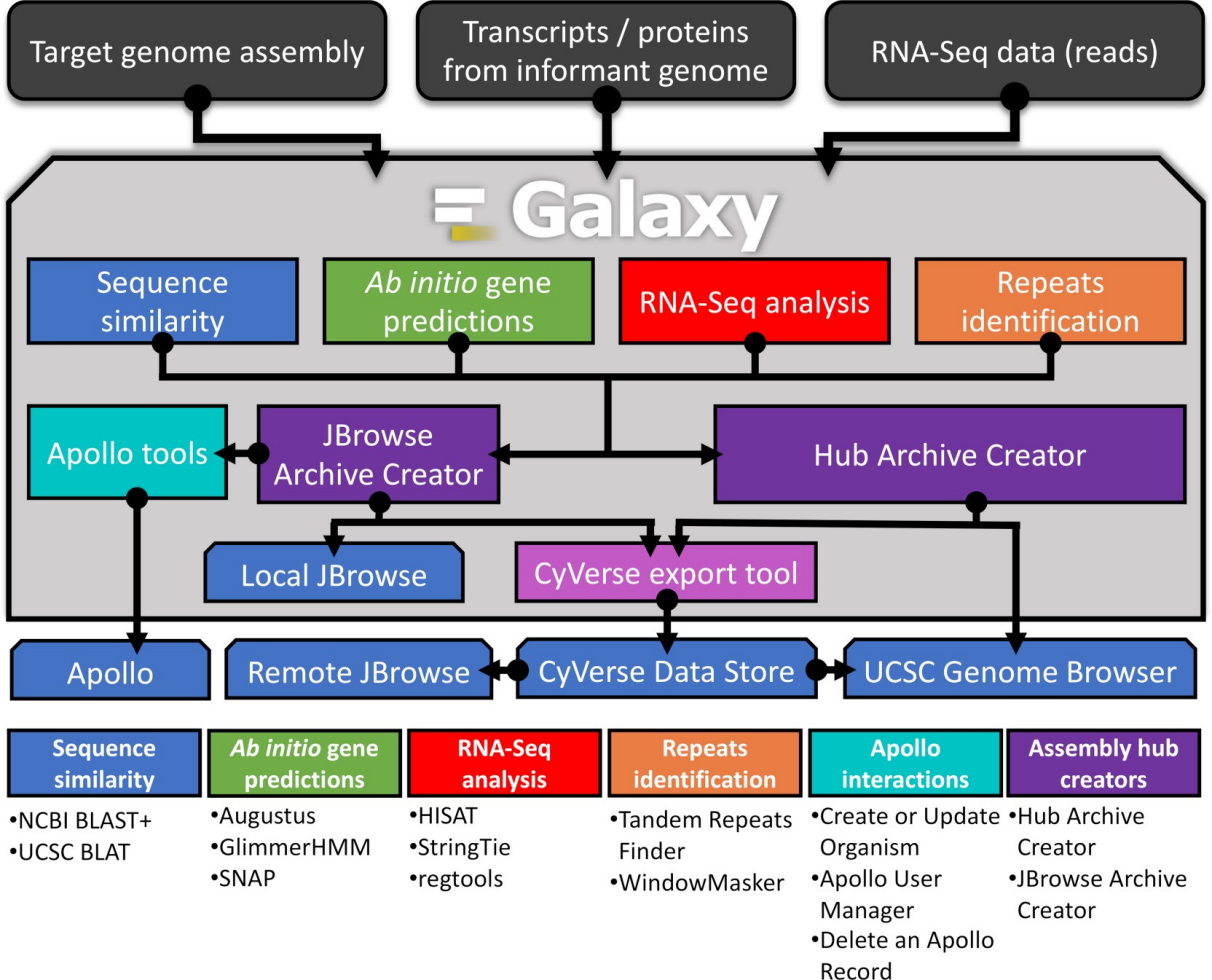
G-OnRamp overview. G-OnRamp is a Galaxy-based platform with analysis workflows that process a target genome assembly, transcripts and proteins from an informant genome, and RNA-Seq data from the target genome to create a genome browser for individual or collaborative annotation. Four sub-workflows (sequence similarity, ab initio gene predictions, RNA-Seq analysis, and repeats identification) run concurrently and generate the data for manual gene annotation. Data produced by the sub-workflows are used by the Hub Archive Creator (HAC) tool to create UCSC Assembly Hubs and by the JBrowse Archive Creator (JAC) to create JBrowse genome browsers. The Apollo interaction tools convert JBrowse genome browsers into an Apollo instance to facilitate collaborative annotations. Genome browsers produced by G-OnRamp can be transferred to the CyVerse Data Store via the CyVerse export tool for long-term storage and visualization. The “Tool Suites” panel (below) lists the primary tools in each sub-workflow and the tools provided by G-OnRamp to create and manage Apollo instances. See [20] and http://g-onramp.org for further details. RNA-Seq, RNA sequencing.

G-OnRamp is a Galaxy-based analysis platform providing a collection of tools and services that enable collaborative genome annotation in an efficient, user-friendly, and web-based environment (http://g-onramp.org; [20]). Galaxy is used across the world by thousands of scientists, and one of its key features is a web-based user interface that anyone can use for complex biological analyses regardless of their computational knowledge. G-OnRamp is configured with tools for sequence similarity searches, gene predictions, RNA-Seq data analysis, and repeat analysis (Fig 3). These tools are combined into multistep workflows that process a target genome assembly and create a UCSC Assembly Hub (which can be viewed at the official UCSC Genome Browser; http://genome.ucsc.edu) or a locally bundled JBrowse instance. G-OnRamp also provides tools to import a JBrowse instance into Apollo to facilitate real-time collaborative genome annotation (https://genomearchitect.readthedocs.io/en/latest/; [10]). In a pedagogical example, an instructor can deploy G-OnRamp, upload the data, run a workflow to generate a JBrowse genome browser for visualization, and use the G-OnRamp Apollo interaction tools to convert the genome browser hub to Apollo for collaborative analysis by students.

#### Apollo interaction tools: Efficiency and crowd management for collaborative annotation

Apollo was included in G-OnRamp as it substantially increases the efficiency of gene annotation. Using Apollo, students can dynamically interact with evidence tracks, selecting the desired exons (by drag and drop) for assembly into a gene model. With effective permission management, annotation can be done separately (different students annotating different genes), iteratively (annotated genes being passed from one student to another), or simultaneously (students collaborate to annotate the same gene at the same time).

To aid permission-driven access control, G-OnRamp provides interaction tools (based on tools developed by the Galaxy community [25]) for managing user accounts and genome assemblies in an Apollo instance. For example, a G-OnRamp administrator can use the “Create or Update Organism” tool to create a new Apollo instance or modify an existing Apollo instance. The Apollo User Manager tool provides fine-grained access controls; an administrator can control the read, write, and export permissions of individual users or groups of users. For example, instructors can use the Apollo User Manager to create accounts for a group of students enrolled in a course, and to limit their access to a subset of the genome assemblies in the Apollo instance.

### Using G-OnRamp in research and education settings

#### G-OnRamp workshops and evaluation

**Fig 4:**
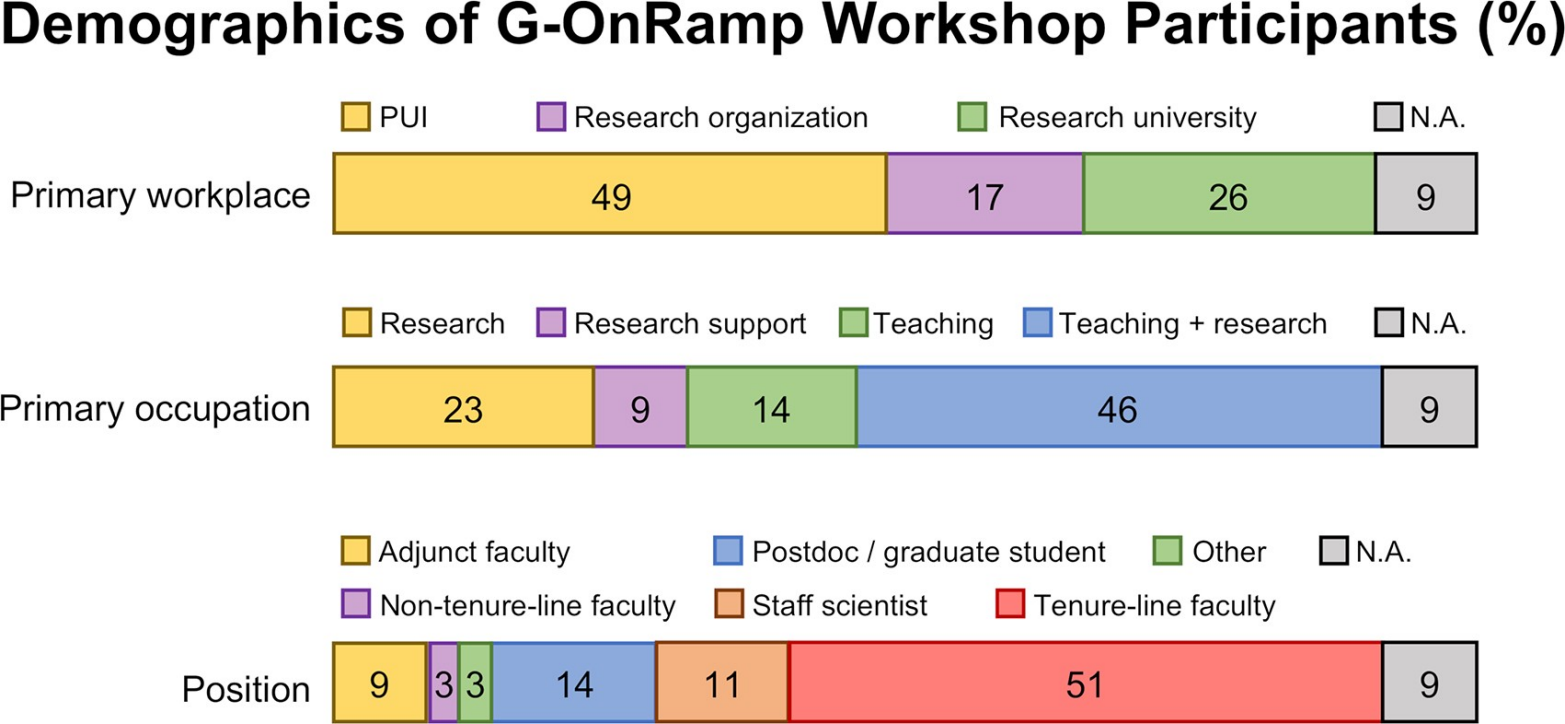
Demographics of G-OnRamp workshop participants. Of the 53 workshop participants eligible, 35 responded to the demographics questions (response rate = 66.0%). Many G-OnRamp workshop participants are tenure-line faculty members who work at PUIs, where they are involved in both teaching and research. Other participants focus mainly on research, either carrying out research or providing research support. PUI, primarily undergraduate institution.

To grow the community of users and better tailor G-OnRamp to their needs, we hosted two beta tester workshops in 2017 and two “train the trainer” workshops in 2018 to introduce researchers and educators to the platform. The goal of these workshops was to familiarize members of the community with G-OnRamp and to solicit feedback. Publicity for the workshops was designed to attract both research scientists and educators with low research support, to demonstrate the potential for mutually beneficial collaboration. These workshops attracted 53 diverse participants from over 40 institutions across the world, demonstrating that G-OnRamp satisfies a need for both researchers and educators alike (Fig 4).

In addition to following a general training curriculum (available at http://g-onramp.org/training) on sample data, attendees were encouraged to bring their own genome assembly for processing and genome browser hub creation. Over 20 publicly available genome browsers were created by workshop participants and the users that tested prototype G-OnRamp versions. Browsers generated during the 2017 and 2018 workshops demonstrate results obtained for genomes with assembly sizes ranging from 70 Mb to 2.1 Gb and with scaffold counts ranging from 53 to 271,888 (Table 1). These genome browsers are hosted on the CyVerse Data Store [26] and are available via the “View Genome Browser” button on the G-OnRamp website (http://g-onramp.org/genome-browsers).

**Table 1:** Publicly available genome browsers.

Target genome (common name)	Genome assembly file size	Number of scaffolds	Informant genome	Number of RNA-Seq samples	Genome browser(s) created
*Aiptasia pallida* (Coral reef)	260 MB	5,065	*Nematostella vectensis*	2	JBrowse + Apollo
*Amazona ventralis* (Hispaniolan parrot)	1.1 GB	18,948	*Gallus gallus*	0	UCSC Assembly Hub
*Amazona vittata* (Puerto Rican parrot)	1.2 GB	16,449	*G*. *gallus*	2	UCSC Assembly Hub
*Bemisia tabaci* (Silverleaf whitefly)	690 MB	19,751	*D*. *melanogaster*	2	UCSC Assembly Hub
*Centrapalus pauciflorus* (Vernonia)	1.2 GB	19,697	*Arabidopsis thaliana*	1	JBrowse
*Chlamydomonas reinhardtii* (Green algae)	113.3 MB	53	*A*. *thaliana*	2	UCSC Assembly Hub
*Fragaria vesca* (Wild strawberry)	240 MB	3,263	*A*. *thaliana*	4	UCSC Assembly Hub
*Ganaspis* sp.1 (Parasitoid wasp)	500 MB	54,394	*D*. *melanogaster*	1	UCSC Assembly Hub
*Schrenkiella parvula* (Saltwater cress)	137 MB	1,457	*A*. *thaliana*	4	JBrowse
*Solenodon paradoxus* (Haitian solenodon)	2.1 GB	40,372	*Erinaceus europaeus*	0	UCSC Assembly Hub
*S*. *paradoxus* (Haitian solenodon)	2.1 GB	3,078	*Homo sapiens*	0	UCSC Assembly Hub
*Spinus cucullatus* (Red siskin)	1.1 GB	26,015	*Taeniopygia guttata*	0	JBrowse and UCSC Assembly Hub
*Taeniopygia guttata* (Zebra finch)	1.26 GB	37,096	*Taeniopygia guttata*	0	UCSC Assembly Hub
*Tetrahymena thermophila* (Ciliate)	155.6 MB	1,464	*Ichthyophthirius multifiliis*	1	UCSC Assembly Hub
*Thalassiosira pseudonana* (Diatoms)	32.8 MB	64	*A*. *thaliana*	2	JBrowse + Apollo
*Thlaspi arvense* (Field pennycress)	539 MB	6,768	*A*. *thaliana*	1	JBrowse and UCSC Assembly Hub
*Xestospongia bocatorensis* (Sponge)	70 MB	271,888	*Amphimedon queenslandica*	8	JBrowse

List of publicly available genome browsers generated with user-submitted data during the 2017–2018 workshops. These and additional G-OnRamp browsers generated by earlier prototypes with user-submitted data can be seen at

http://g-onramp.org/genome-browsers.

Abbreviation: RNA-Seq, RNA sequencing

#### G-OnRamp features

Feedback collected from participants after each workshop was used to determine priority areas for improvements in documentation, performance and scalability of the workflows, accessibility of the user interface, and quality-of-life improvements to extant tools. For example, the 1.1 release of G-OnRamp includes requested improvements to Galaxy’s support for Augustus, a tool that performs comparative gene prediction [27], enabling users to limit the genomic range to search or to add extrinsic “hints” for improved search specificity. Beyond this, the 1.1 release of G-OnRamp features the latest (as of this writing) versions of Galaxy (19.05), Apollo (2.4.1), and JBrowse (1.16.6). A more complete list of features is provided in Table 2.

**Table 2:** Features: G-OnRamp provides….

**Processing/Analysis**
The UCSC HAC, a tool to create genome browser archives for display with the UCSC browser
The JAC, a tool to create JBrowse genome browsers with Galaxy
An RNA-Seq analysis sub-workflow to process and visualize RNA-Seq data
A BLAT alignment sub-workflow to align transcript sequences from an informant genome to the target genome
Tools to identify repeats using WindowMasker within Galaxy
**Input/Data Acceptance**
Default workflows that accept genome assemblies in fasta format, RNA-Seq data in fastqsanger format, transcripts from informant genomes in GenBank or fasta formats, and proteins from informant genomes in fasta format
Added tools to facilitate the incorporation of results from additional gene predictors and RNA-Seq alignment tools (e.g., bigWig and BAM files) into the genome browsers produced by G-OnRamp
An extended Augustus tool Galaxy wrapper, exposing more functionality (e.g., ability to specify search range or add extrinsic hints)*
An improved HAC and the JAC tools (e.g., bug fixes, added support for new track types and custom tracks)*
**Annotation Support**
Tools and a workflows to create Apollo instances from JBrowse genome browsers, and to support collaborative genome annotation using Apollo
Improved role-based access control in Apollo to facilitate collaborative annotation in educational settings*
Reporting features for instructor roles in Apollo to enable faculty to monitor student annotation progress*
**General Ease of Use**
The G-OnRamp website (http://g-onramp.org), which hosts documentation, training resources, and previously processed data
A CyVerse interaction tool to facilitate the data import and export between G-OnRamp and the CyVerse Data Store
JBrowse improvements to display tblastn alignments that span larger genomic regions*
Optimized search index strategies for feature names and descriptions in JBrowse to reduce the number of index files (e.g., Tabix-indexed GFF3 files)*
The ability to look up gene predictions and the BLAST and BLAT alignments by name (e.g., RefSeq accession numbers) and by description
Links to external database records (e.g., at NCBI, FlyBase) for the tblastn and BLAT alignment tracks
Improved organization, grouping, and labeling of evidence tracks on UCSC Assembly Hubs
Comprehensive training materials based on feedback from the participants of the G-OnRamp beta tester workshops
**Deployment**
Automated local and cloud (Amazon Elastic Cloud Compute [EC2]) deployments of G-OnRamp with GalaxyKickStart—an Ansible playbook for deploying production Galaxy servers
A G-OnRamp image deployable via CloudLaunch (https://launch.usegalaxy.org) to enable users with limited technical expertise to run G-OnRamp on the cloud (Amazon EC2)
A G-OnRamp image deployable via the Amazon Web Services (AWS) EC2 console

List of major features developed for the G-OnRamp platform and improvements made to various software components. Feature and improvement development was driven predominantly by user feedback, most of which was gathered from attendees of our biannual G-OnRamp workshops. While improvements were made throughout the cycle of G-OnRamp development, feedback from these events was a valuable aid to prioritization.

*Features or improvements that were developed for component services of G-OnRamp that are now generally available for those services.

Abbreviations: HAC, Hub Archive Creator; JAC, JBrowse Archive Creator; RNA-Seq, RNA sequencing

**Fig 5:**
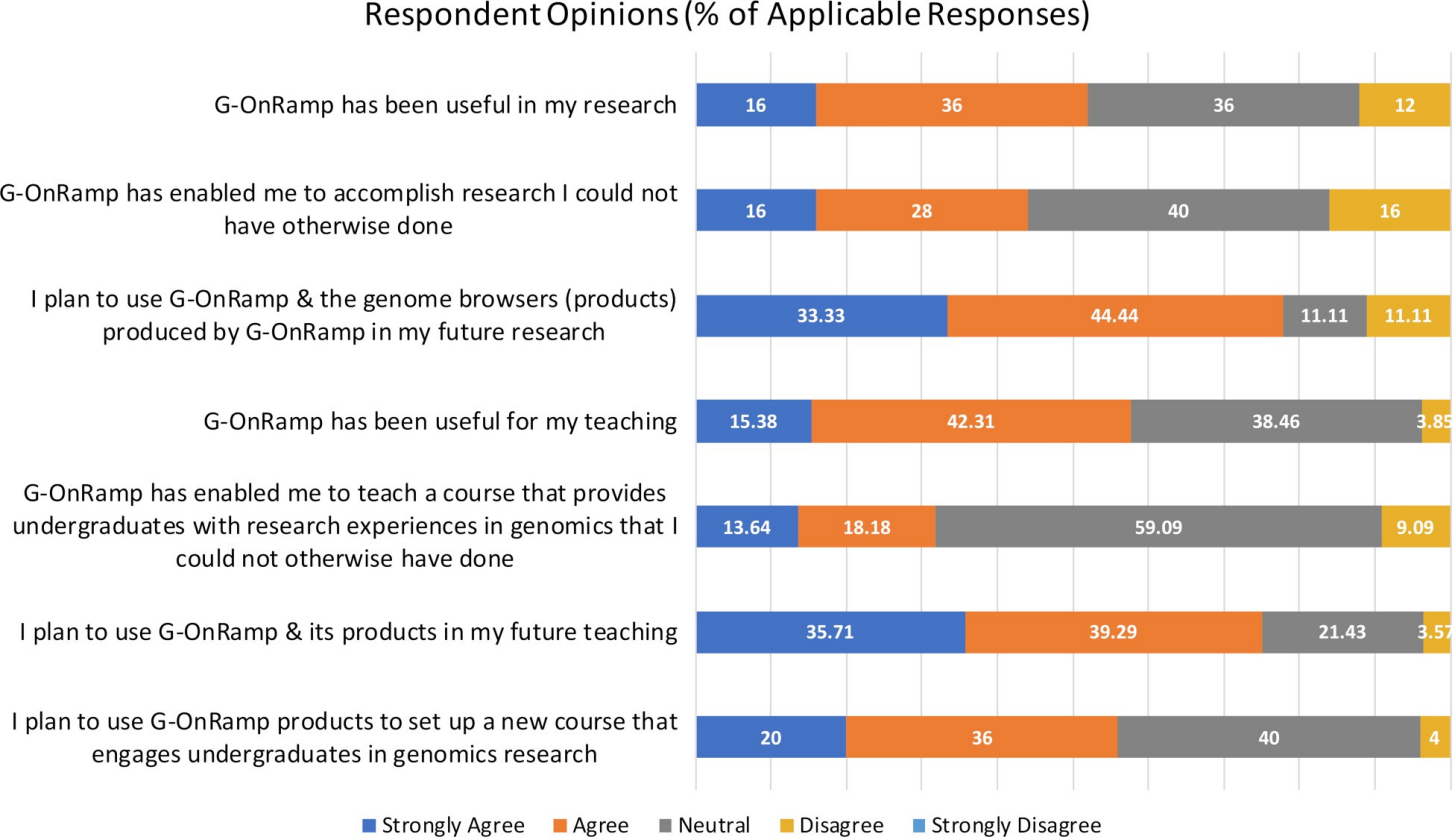
Survey responses on the utility of G-OnRamp. An anonymous survey asked respondents (*N* = 35 of 53 eligible) to check “strongly agree,” “agree,” “neutral,” “disagree,” or “strongly disagree.” Participants ranged from those whose primary occupation is teaching to those managing a research support service (see Fig 4). Consequently from 20% to 38% of the participants checked “not applicable” for any given statement; these responses were removed before percentages were calculated. Overall, participants reported that G-OnRamp facilitates both research and teaching.

Based on the results from an anonymous survey of G-OnRamp workshop participants, we find that the overall response by users has been very good (see S1 Text for a copy of the Institutional Review Board [IRB] approval memo). Both researchers and educators reported that G-OnRamp has facilitated their work (Fig 5). A majority of the respondents found G-OnRamp useful in their research and/or teaching and planned to continue to use it, including setting up new student research courses.

#### Using G-OnRamp in a CURE: Examining lipid synthesis pathways in parasitoid wasps

As discussed above, many bioinformatics educators have found that a genome annotation project is a good way to introduce students to genomics while providing a research experience. This can be implemented as a one-semester CURE or as a shorter unit to provide students with an introduction to research.

Many genomics projects that can benefit from careful manual annotation will be focused on a limited set of genes. Because these genes of interest are commonly defined by a shared functional annotation or membership in a specific pathway, they are likely to be dispersed throughout the genome. In the case study presented here, the project is focused on the evolution of lipid synthesis pathways in parasitoid wasps, and so the genes of interest are defined based on their predicted functions rather than their genomic locations. This case was used to test the acceptability and utility of G-OnRamp products in the undergraduate lab.

**Fig 6:**
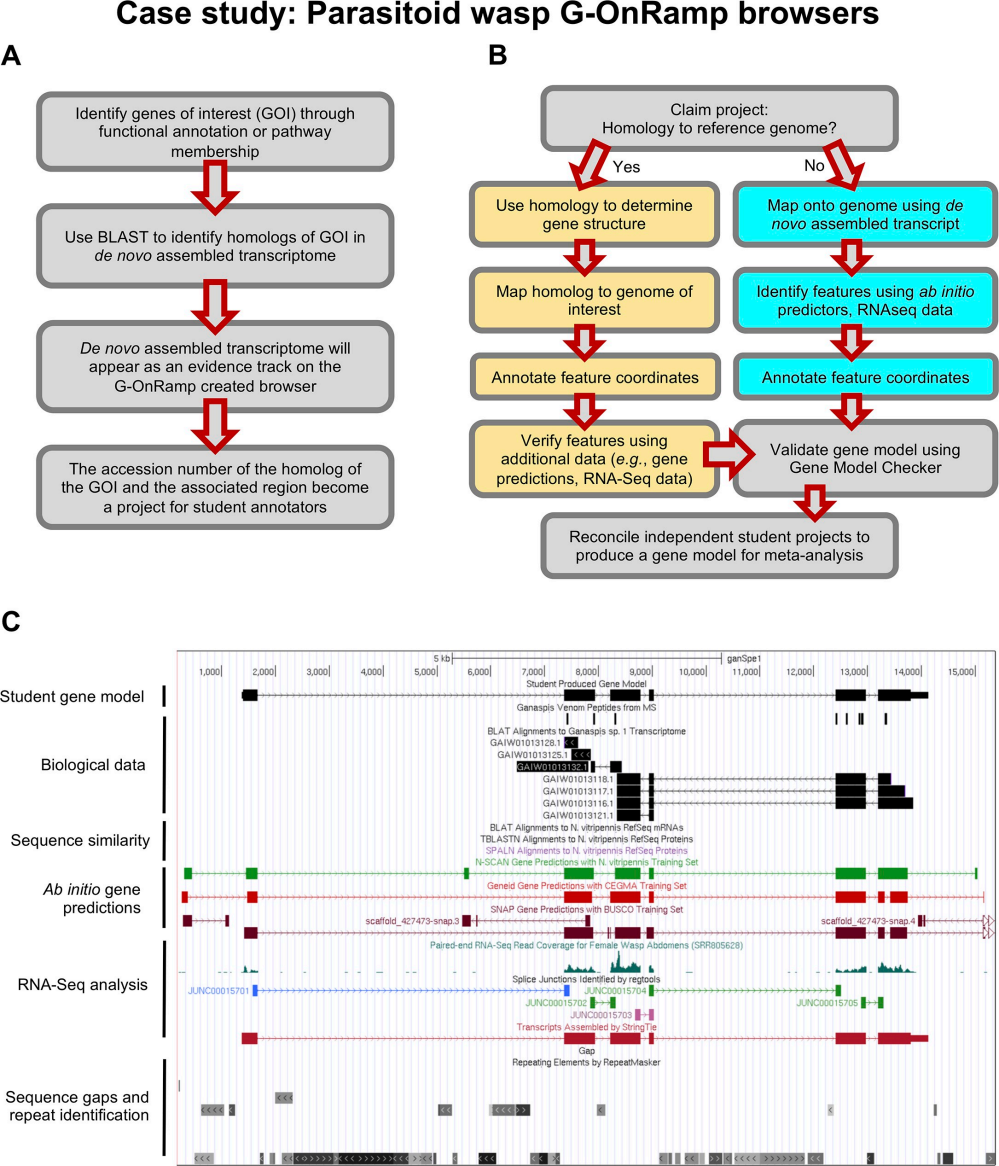
Case study: Annotation using parasitoid wasp G-OnRamp browsers. A. The workflow for identifying genes of interest and creating student annotation projects based on G-OnRamp browsers. B. The student annotation workflow. Students are assigned a project and will then work through either of the two sub-workflows depending on homology of the gene of interest to the reference genome. Boxes in yellow define the sub-workflow for genes with homology to the reference genome; cyan boxes define the sub-workflow for genes lacking homology to the reference genome. C. An example student annotation of a gene with no homology to the reference genomes (*D*. *melanogaster* or *Nasonia vitripennis*). Survey respondents identified lack of homology to an informant genome as one of the main challenges in annotating new species. RNA-Seq, RNA sequencing.

Fig 6A illustrates the workflow underlying the creation of student annotation projects, in which the approximate locations of the genes of interest are identified in the newly sequenced genomes and assigned as student projects. Fig 6B outlines the approach taken by the student annotator, which is predicated on sequence similarity between the gene of interest in the target genome and genes from an informant genome. The difficulty of the student project primarily depends on the result of the homology search. Modifications of this workflow will be appropriate for other projects, depending on the types and quality of data available for the genomes under study.

A gene that aligns to an ortholog in a well-studied informant species will not be very difficult for an undergraduate to annotate, while the absence of orthologs will create a challenge. If the gene of interest has significant similarity to a gene in the informant genome, then the student annotator would construct the most parsimonious gene model compared to its putative ortholog in the informant genome. Otherwise, the student annotator would use RNA-Seq data to construct the gene model. Instructors can prescreen projects to select those at the appropriate level of difficulty for their students (see S2 Text).

Fig 6C illustrates an example of a student annotation of a gene that has diverged from the informant genomes (*N*. *vitripennis* and *D*. *melanogaster*) such that homology data are not available. The student annotator has to construct a gene model based on other lines of evidence, such as proteomics data, RNA-Seq data (e.g., read coverage, de novo transcriptome assembly), and ab initio gene predictions. The flexibility of the genome browsers produced by G-OnRamp, and the annotation workflow described above, have facilitated annotation in this case, and should make comparative genomics more accessible for use in the classroom, creating opportunities to study other newly sequenced genomes.

#### Evaluation of G-OnRamp in a CURE: Parasitoid wasps

**Fig 7:**
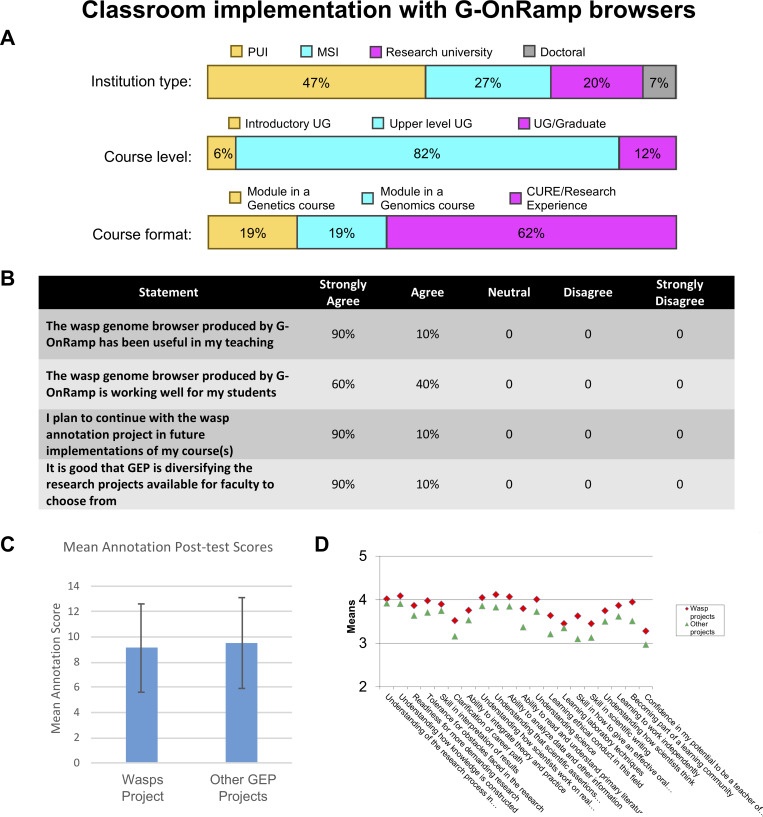
Using G-OnRamp in a CURE. Classroom implementation with G-OnRamp genome browsers. A. Implementations of the parasitoid wasp project during 2017–2018 and 2018–2019 characterized by institution type (*n* = 15), course level (*n* = 16), and course format (*n* = 16). B. Results from a survey of faculty who have used a G-OnRamp–generated genome browser in a course. Participants were asked to respond on a 5-point Likert Scale with NA as an option; of the 14 faculty responding to this portion of the survey, the four checking “NA” for these questions were removed before calculating percentage responses, giving *n* = 10. Responses are shown by percentage of respondents. C. Mean annotation post-course test scores: The mean for the wasp group is 9.1 (*N* = 173; SD = 3.6) and the mean for the other GEP students is 9.5 (*N* = 1,185; SD = 3.5). The difference is not significant (bars represent the means; error bars represent one standard deviation). D. Responses to the SURE survey questions: The means for the wasp project students are in red (*N* ranges from 181 to 195, as some students did not answer all questions) and the means for the other GEP students (working in *Drosophila*) are in green (*N* ranges from 1,200 to 1,270). For some items, the wasp group scores significantly higher than the comparison group; however, these results should be interpreted with caution, given the small sample size. CURE, Course-based Undergraduate Research Experience; GEP, Genomics Education Partnership; NA, Not Applicable; PUI, primarily undergraduate institution; MSI, Minority-Serving Institution; SURE, Survey of Undergraduate Research Experiences; UG, undergraduate.

In this pilot implementation of a CURE project using genome browsers generated by G-OnRamp, 15 faculty from the GEP designed CUREs for their students based on the parasitoid wasp research project. These faculty members came from diverse schools (Fig 7A; a full list of faculty with their schools is given in the Acknowledgments). The courses ranged from freshman/sophomore level to those that provided graduate credit. The majority were structured as a research experience. Responses from an anonymous survey show that most faculty found that the wasp genome browser produced by G-OnRamp worked well for their students and was generally useful in teaching (Fig 7B). Faculty members who responded to the survey all planned to continue involving their students in the parasitoid wasp project the following year, and all applauded the effort by the GEP/Galaxy partnership to support genomics research broadly.

Past GEP assessments have shown that students who have participated in the GEP research projects exhibit greater knowledge gains about the fundamentals of eukaryotic genes and genomes compared to students who did not participate in the GEP research projects [21, 22]. To evaluate the efficacy of using G-OnRamp genome browsers in educational settings, direct assessment of the students engaged in a parasitoid wasp CURE was obtained by comparing the responses of this group to those of GEP students as a whole, looking at pooled data from 2017–2018 and 2018–2019. The post-course quiz scores for the students who have participated in the wasp research project show no significant difference compared to students who have participated in the *Drosophila* Muller F element project (Fig 7C). This result indicates that using the genome browsers produced by G-OnRamp is as effective as using the GEP mirror of the UCSC Genome Browser in teaching students the fundamentals of eukaryotic genes and genomes. Interestingly, there is a small increase in the responses to the Survey of Undergraduate Research Experiences (SURE) survey questions [28], which ask students to self-report perceived gains in the understanding of how science is done and their acquisition of research skills (Fig 7D). This suggests that G-OnRamp can increase student and faculty enthusiasm for genomics research by enabling a variety of projects.

Eventually, we hope to see multiple collaborative annotation projects that would allow all faculty to participate in a project according to their research interests. A number of studies have demonstrated benefits from engaging students in CUREs [29, 30], and genomics research is generally less expensive and easier to manage in an academic-year course than a wet bench project. Several other projects that engage students in a genomics CURE can be accessed from the home page of the Genomics Education Alliance (GEA; https://gea.qubeshub.org).

#### Using G-OnRamp on your own

G-OnRamp is freely available on GitHub under an Academic Free License version 3.0 (https://github.com/goeckslab/GOnRampKickStart). To help users get started, we also provide virtual machines with G-OnRamp preinstalled for use on a local computer and in cloud computing environments, thereby enabling the use of G-OnRamp worldwide. Steps for acquiring and deploying G-OnRamp, like the platform itself, minimize technical complexity and accelerate data analysis activities. The two principal methods of deployment meet different user needs: (1) a VirtualBox virtual appliance for small-scale local testing and training and (2) an Amazon Machine Image (AMI) for cloud-based production deployments. Users can launch the G-OnRamp AMI on Amazon Web Services (AWS) via either the CloudLaunch web application or the AWS Marketplace (https://launch.usegalaxy.org/; Table 3). See the “G-OnRamp deployment options” page on the G-OnRamp web site for detailed instructions (http://g-onramp.org/deployments). Free training materials (presentations, walkthroughs, and exercises) developed for the 2017–2018 workshops provide sufficient detail to enable novices to get started on their own (http://g-onramp.org/training). Users who have questions about Galaxy can contact members of the Galaxy Training Network (https://galaxyproject.org/teach/gtn/) from around the world or post questions on the Galaxy Community Help forum online (https://help.galaxyproject.org/).

**Table 3:** Deployment options.

Deployment Option	URL	Notes	Documentation
Virtual Machine (VM) Image	https://wustl.box.com/v/g-onramp-vm-v1-1	For local testing/training with G-OnRamp; not sufficiently performant for high-scale analysis. However, the VM can be used for smaller genomes, depending on the resources allocated to the VM	https://wustl.box.com/s/vnz0z6a9rsgglua10phlpu1uztwpre4x
AWS via CloudLaunch	https://launch.usegalaxy.org/catalog	For any level of analysis; instance resources configurable by the user. Select “G-OnRamp” from the Appliance Catalog to launch on AWS without using the console	https://wustl.box.com/s/ohg7lsvjbsc601rtddyej1ud61tlo7qu
AWS Marketplace	https://console.aws.amazon.com/ec2/	For any level of analysis; instance resources configurable by the user. When launching an instance, search for “G-OnRamp” from “Community AMIs”	https://wustl.box.com/s/5we1dit8z3yaf8t5h520zgmeakutn77o

Alternative G-OnRamp deployment methods, their strengths and weakness, and relevant documentation.

Abbreviations: AMI, Amazon Machine Image; AWS, Amazon Web Services

For more fine-grained control of the installation and launch of G-OnRamp, the scripts used to create the two principal deployment options are open source and available on GitHub (https://github.com/goeckslab/gonrampkickstart). This option provides much greater control but comes with additional complexity that requires technical expertise. For more complex deployment configurations within the AWS infrastructure, a G-OnRamp image can be found under “Community AMIs” when launching an Elastic Cloud Compute (EC2) instance.

## Conclusion

The importance and efficacy of providing undergraduates with a research experience is widely accepted. While it is difficult to identify the impact of research per se [31], students engaged in a CURE are reported to be both retained in the sciences and to graduate within six years at a higher frequency than matched students who do not have this experience [29]. CUREs in bioinformatics have many advantages, both practical and pedagogical: infrastructure costs are low (requires only computers and internet connectivity), and there is a large and growing pool of publicly available data, along with tools to manage and analyze that data (e.g., Galaxy, CyVerse). CUREs in bioinformatics also lend themselves to peer instruction, an important multiplier, as students can collaborate on their own schedule; no physical lab is required, access is 24/7, and there are no lab safety issues. Perhaps most important, student mistakes are inexpensive in time and money, as the annotation process can be quickly reiterated, problems explored, and investigations taken to the next level. Recognizing these advantages, a growing number of faculty groups have emerged over the last decade to organize CUREs that include collaborative genome annotation [8,32–34]. Recently, several of these groups have come together to form a GEA (https://gea.qubeshub.org), which seeks to support this effort by creating a common, well-maintained platform with common curriculum and tools [35]. The advent of cloud computing enables researchers and educators with limited local compute resources to perform large-scale bioinformatics analyses. Many major cloud platforms provide free credits for educators to engage students in research (e.g., the AWS Educate program; https://aws.amazon.com/grants). Starting from an assembled genome, G-OnRamp removes one bottleneck to CURE growth in bioinformatics by facilitating creation of the genome browsers needed for collaborative genome annotation projects. The G-OnRamp survey results and the parasitoid wasp pilot project have shown G-OnRamp to be a useful tool for researchers and educators alike.

## Supporting information

S1 Text IRB Approval Memo. The anonymous G-OnRamp surveys were reviewed and approved by the Washington University in St. Louis Institutional Review Board (IRB ID # 201902059); this is a copy of the IRB approval memo. IRB, Institutional Review Board.
(PDF)

S2 Text Gene Difficulty Rubric—Wasp Project. A rubric for estimating the difficulty of the wasp annotation projects based on multiple factors, including the level of sequence similarity with proteins and transcripts from the informant genome, availability of RNA-Seq data, gaps in the genome assembly, estimated number of isoforms and exons, and the amount of overlap between the gene predictions and the other lines of evidence. RNA-Seq, RNA sequencing.
(DOCX)
