# Supplementary material for: G-OnRamp: Generating genome browsers to facilitate undergraduate-driven collaborative genome annotation
Source: PLoS Comput Biol. 2020 Jun 4;16(6):e1007863. doi: 10.1371/journal.pcbi.1007863 (PMC7272004; doi:10.1371/journal.pcbi.1007863)
Supplement: S2 Text — A rubric for estimating the difficulty of the wasp annotation projects based on multiple factors, including the level of sequence similarity with proteins and transcripts from the informant genome, availability of RNA-Seq data, gaps in the genome assembly, estimated number of isoforms and exons, and the amount of overlap between the gene predictions and the other lines of evidence. RNA-Seq, RNA sequencing. (DOCX) [file pcbi.1007863.s002.docx]

Gene Difficulty Rubric – Wasp Project

Overall Goal: Develop a rubric for estimating the difficulty of genes to annotate for the wasp project, with an increasing scale of 1-3 for difficulty. My idea is to rate each gene on each of these criteria and then average the score as an overall rating.

Rating Criteria:

Homology *weighted double

Strong homology to *Drosophila* or *Nasonia* 1

Weak homology 2

No homology 3

Notes on homology: From what I’ve seen, not having homology seems to be the factor that can make annotation the most difficult. Is it worth putting a higher value for “no homology” genes to weight it? Consensus among wasp genome pioneers is yes, and that this is the biggest factor in determining difficulty in these species.

RNAseq Data *weighted double

Yes 1

Yes, but difficult to interpret 2

No 3

Notes on RNAseq: For the “Yes, but difficult to interpret” category I was thinking about those genes where StringTie isn’t able to make a prediction, either due to a fairly low expression level or a complex region (overlapping genes, etc.)

Preliminary Assembly (=Transcriptome track) *weighted double

Looks mostly complete 1

Split across multiple predictions 2

Incomplete 3

Notes of Preliminary Assembly: For this I’m thinking about the Transcriptome track that was derived from the de novo assembly of the RNAseq data before we had a genome to align it to. I’ve noticed several genes where all of the gene is accounted for in the data, but it was originally predicted as separate transcripts, so the student needs to combine two or three to get the whole gene. I also found a few where only parts of the gene were originally predicted so students need to use other data to fill in the missing info.

Sequence Gaps

No 1

In the Introns or UTRs 2

In the CDS Disqualify gene

Notes of Sequence Gaps: The improved genomes should be available soon, but for now some of the scaffolds have sequence gaps. If the gaps are within introns or the UTR it can make it more difficult but still possible to come up with a decent gene model (there is value for the project to have CDS only models if the complete model isn’t annotatable).

Splice Forms

1 1

2 or 3 2

4+ 3

Exons

1-2 1

3-8 2

9+ 3

Gene Predictors

Good 1

Partial 2

Useless 3

Notes on Gene Predictors: Good refers to genes with overlap between multiple gene predictors, Partial refers to genes with minimal overlap, Useless refers to genes without predictions or where the predictors all disagree

Scoring metric ((2*(Homology + RNAseq + Transcriptome track)) + (Gaps + Splice forms + Exons + Predictors))/2

Rating: Genes will be rated with increasing difficulty based on the scoring metric. Once several genes have been scored, the distribution of scores will determine the thresholds for the ratings.

Sample genes:

GAIW01010771.1

GAIW01011364.1

GAIW01011026.1

GAIW01011442.1

GAIW01013132.1

GAIW01015059.1
